# Gallstone Classification Using Random Forest Optimized by Sand Cat Swarm Optimization Algorithm with SHAP and DiCE-Based Interpretability

**DOI:** 10.3390/s25175489

**Published:** 2025-09-03

**Authors:** Proshenjit Sarker, Jun-Jiat Tiang, Abdullah-Al Nahid

**Affiliations:** 1 Electronics and Communication Engineering Discipline, Khulna University, Khulna 9208, Bangladesh; 2 Centre for Wireless Technology, CoE for Intelligent Network, Faculty of Artificial Intelligence & Engineering, Multimedia University, Persiaran Multimedia, Cyberjaya 63100, Selangor, Malaysia

**Keywords:** gallstone, machine learning, random forest classifier, Sand Cat Swarm Optimization, SHAP, DiCE

## Abstract

Gallstone disease affects approximately 10–20% of the global adult population, with early diagnosis being essential for effective treatment and management. While image-based machine learning (ML) models have shown high accuracy in gallstone detection, tabular data approaches remain less explored. In this study, we have proposed a Random Forest (RF) classifier optimized using the Sand Cat Swarm Optimization (SCSO) algorithm for gallstone prediction based on a tabular dataset. Our experiments have been conducted across four frameworks: only RF without cross-validation (CV), RF with CV, RF-SCSO without CV, and RF-SCSO with CV. Only RF without CV model has achieved 81.25%, 79.07%, 85%, and 73.91% accuracy, F-score, precision, and recall, respectively, using all 38 features, while the RF with CV has obtained a 10-fold cross-validation accuracy of 78.42% using the same feature set. With SCSO-based feature reduction, the RF-SCSO without and with CV models have delivered a comparable accuracy of 79.17% and 78.32%, respectively, using only 13 features, indicating effective dimensionality reduction. SHAP analysis has identified CRP, Vitamin D, and AAST as the most influential features, and DiCE has further illustrated the model’s behavior by highlighting corrective counterfactuals for misclassified instances. These findings demonstrate the potential of interpretable, feature-optimized ML models for gallstone diagnosis using structured clinical data.

## 1. Introduction

**Figure 1:**
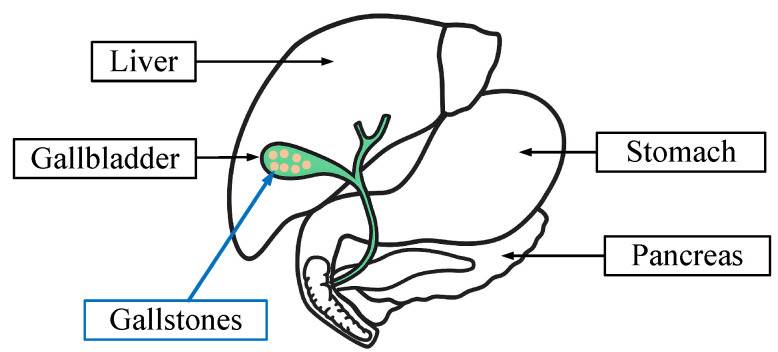
Gallstones in the gallbladder.

Gallstones are solid and crystalline deposits in the gallbladder or bile ducts caused by high levels of cholesterol or bilirubin in bile [1,2]. About 10–20% of adults worldwide have gallstones. Gallstones are mainly classified by composition: over 90% are cholesterol stones, and under 10% are pigment stones (black or brown), and by location, either in the gallbladder or bile ducts (extrahepatic or intrahepatic) [1]. Gallstones develop when bile contains excess cholesterol or lacks enough bile salts, combined with poor gallbladder movement. Common risk factors are female gender, pregnancy, aging, obesity, specific ethnic groups, unhealthy eating habits, quick weight loss, and certain illnesses [2]. In Western countries, most gallstones (75–80%) are cholesterol stones, meaning they are made of more than 50% cholesterol [3]. The rest are pigment stones, made of less than 30% cholesterol, and these are further divided into black pigment stones (10–15%) and brown pigment stones (5–10%). Gallstone diagnosis usually begins with assessing pain in the upper right abdomen, although this can also be caused by other conditions [4]. Blood tests such as Complete Blood Count (CBC), Liver Function Test (LFT), and measurements of enzymes like lipase and amylase support the diagnosis. However, imaging techniques are the most accurate, with Ultrasonography (US) being the gold standard due to its high sensitivity and specificity. In unclear cases, additional imaging methods such as Computed Tomography (CT), Magnetic Resonance Cholangiopancreatography (MRCP), or Endoscopic Retrograde Cholangiopancreatography (ERCP) are used. Figure 1 shows the presence of gallstones in the gallbladder.

Early detection of gallstones enables the initiation of timely treatment and significantly reduces the burden on patients. In this context, machine learning (ML) techniques offer a promising solution. Previous studies have demonstrated the effectiveness and growing acceptance of ML methods for accurate gallstone detection and localization. Bozdag et al. have used Content-Based Image Retrieval (CBIR) systems to classify gallbladder diseases, including gallstones [5]. They have achieved an accuracy of 94.4%. Wang et al. have proposed an ultrasound (US) image-based model that has gained an Area under the Curve (AUC) of 0.995 using the XGBoost-US radiomics model [6]. Obaid et al. have used images to identify gallbladder diseases with an accuracy of 98.35%, where the gallstone classification accuracy has been recorded as 98% [7]. Pang et al. have used the You Only Look Once (YOLO) algorithm on CT scan images to classify gallstones and have succeeded in attaining 86.5% accuracy [8]. Hong et al. have used the Random Forest (RF) classifier on US images and shown 96.33% accuracy [9]. The previously discussed research studies have primarily focused on image-based approaches. However, unlike the previously mentioned image-based approaches, Esen et al. utilized a tabular dataset and achieved an accuracy of 85.42% in predicting the presence of gallstones using a Gradient Boosting Classifier [10].

This research focuses on evaluating the performance of the Sand Cat Swarm Optimization (SCSO) algorithm in combination with the Random Forest (RF) classifier for gallstone classification using a tabular dataset. We have used a publicly available dataset, Gallstone, available on the UC Irvine Machine Learning Repository. The dataset contains 39 columns (38 features + a binary target class) and 319 subjects. The detailed description of the dataset is given in the dataset subsection. Most of the previous studies [5,6,7,8,9] have relied on image-based approaches, which typically involve complex models and slower identification processes compared to tabular dataset-based models. However, Esen et al. [10] proposed a tabular dataset approach using 38 features. This high number of features presents practical limitations, as it requires extensive clinical testing, which can be both time-consuming and costly. Overall, the existing studies face several limitations, including high model complexity, a large number of required features, and a lack of in-depth analysis, particularly regarding the nature of misclassification. An ML model that does not provide insights into its decision-making process is often considered a “black box”. In previous studies, most models have remained black boxes, as they failed to explain the rationale behind individual predictions, such as why a particular case was classified as gallstone positive or negative.

To overcome the limitations of prior studies, we have proposed a feature-reductive ML approach leveraging metaheuristic algorithms (MHAs). MHAs are optimization techniques inspired by natural phenomena, such as animal hunting strategies and particle interactions, and are widely applied for both feature selection and hyperparameter tuning [11,12]. Previous studies have shown the acceptance of different types of MHAs on medical datasets for feature selection and hyperparameter tuning. Stephan et al. have used an artificial bee colony with whale optimization on breast cancer datasets to improve classification task [13]. Li et al. (2021) have demonstrated that the integration of particle swarm optimization with neural networks can significantly improve classification performance in medical data [14]. Previous studies have shown the multidimensional applicability of the SCSO algorithm in diverse domains, such as biomedical applications [15] and cancer classification [16]. Ref. [17,18] have used the gray wolf optimization algorithm for classifying heart-related issues such as Coronary Artery Disease and Heart Failure.

In this research, we have focused on assessing the effectiveness of MHAs in identifying gallstone cases using a minimal set of diagnostic features, thereby reducing the need for extensive and costly clinical testing. Moreover, we have prioritized reducing the computational complexity and execution time of the models to enhance their practicality in real-world clinical settings. To address the black-box nature of many ML models, we have incorporated explainable artificial intelligence (XAI) techniques. Specifically, we employed Shapley Additive Explanations (SHAP) and Diverse Counterfactual Explanations (DiCE) to interpret model decisions and provide transparency into feature contributions and prediction logic. Recent studies on medical health care have proven the integration of these XAI algorithms. Ref. [19] has used SHAP and DiCE in their research to enhance the mild cognitive impairment and Alzheimer’s disease diagnosis. Su et al. have visualized the feature importance using SHAP, highlighting which features are most influential to the model [20]. AlJalaud et al. have implemented DiCE on four different datasets, including the breast cancer dataset [21]. In addition to model interpretation, we have conducted an in-depth analysis of misclassified instances, aiming to understand the underlying reasons for incorrect predictions and explore how these can be corrected through model refinement. To ensure the robustness and generalizability of our approach, we have implemented a customized 10-fold cross-validation strategy, allowing the model to be evaluated comprehensively on unseen and practical data scenarios. The performance of the model has been evaluated using accuracy, F1-score, precision, recall, Area Under the Curve (AUC), and Receiver Operating Characteristic (ROC) curve. So, considering these, our main contributions are as follows:

■ We have used the Sand Cat Swarm Optimization (SCSO) algorithm for simultaneous feature selection and hyperparameter tuning within the Random Forest (RF) framework for gallstone classification;
■ We have introduced a metaheuristic-based optimization pipeline that reduces the number of features while improving classification accuracy and computational efficiency in clinical usages;
■ The proposed model incorporates interpretable AI techniques, including SHAP for detailed feature contribution analysis and DiCE for generating counterfactual explanations, enabling a clinically meaningful understanding of predictions;
■ In this study, the model’s performance has been systematically evaluated across multiple metrics, including accuracy, F1-score, precision, recall, AUC, ROC, and execution time, demonstrating both effectiveness and efficiency.

The remaining portion of this research study has been organized as Materials and Methods, Results, Discussion, and Conclusion. In Section 2, we have described the dataset, ML algorithm, and the development of the proposed frameworks. In Section 3, the findings of the research work have been presented and explained. Then we have compared our models with existing works in Section 4. Finally, we have concluded this research work, highlighting the key findings in Section 5.

## 2. Materials and Methods

**Figure 2:**
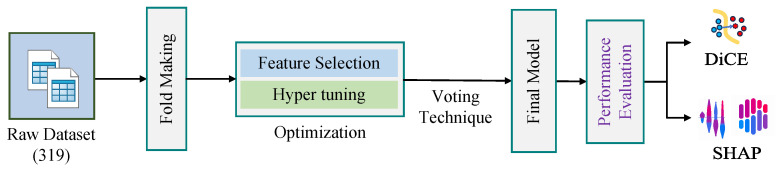
Research methodology.

In this section, we have presented the dataset used in this work. We have also described the ML algorithms used in this work, such as SCSO, RF, SHAP, and DiCE, including the equations of evaluation metrics. Figure 2 shows our overall methodology for the research work.

### 2.1. Dataset

In this study, we utilized the gallstone dataset publicly available from the UC Irvine Machine Learning Repository (Gallstone-UCI dataset) [10], accessible at https://archive.ics.uci.edu/dataset/1150/gallstone-1 (accessed on 19 July 2025). This dataset comprises 319 records (161 gallstone patients and 158 healthy controls) collected from the Internal Medicine Outpatient Clinic of Ankara VM Medical Park Hospital, each represented by 38 quantitative attributes in addition to a binary target label indicating the presence (0) or absence (1) of gallstones. The dataset is fully complete, containing no missing values, and therefore does not require any imputation. Additionally, all categorical and binary features are already labeled and encoded, eliminating the need for further preprocessing. The dataset comprises 161 patients with gallstones and 158 healthy controls, reflecting a balanced class distribution. We have prepared a 10-fold dataset, ensuring proper shuffling to facilitate the effective implementation of the machine learning algorithms. In each fold, the dataset was split into two distinct sets: 70% for training and 30% for testing.

**Figure 3:**
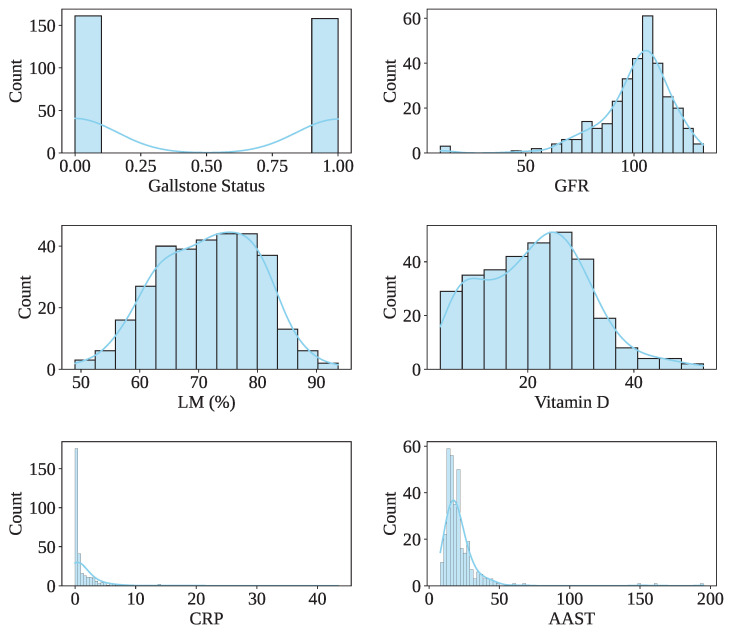
Distribution of dataset values of some features, including the target class.

These attributes are derived from routine clinical laboratory evaluations and include hematological parameters (e.g., white blood cell count, red blood cell indices, platelet count), hepatic function biomarkers (e.g., alanine transaminase [ALT], aspartate transaminase [AST], alkaline phosphatase [ALP], total and direct bilirubin), lipid profile components (e.g., total cholesterol, HDL, LDL, triglycerides), and renal function indicators (e.g., creatinine, urea), among others. Demographic variables such as age and gender are also included. Table 1 presents the description of the Gallstone-UCI dataset. The dataset has 161 gallstone patients and 158 healthy controls, indicating a balanced dataset. In the dataset, 91.54% (292) of people’s ages vary from 30 to 70, indicating that most of the people are middle-aged. The male and female patient ratio is 162:157. Figure 3 shows the data value distribution of the rest of some features, including the target class of the dataset.

**Figure 4:**
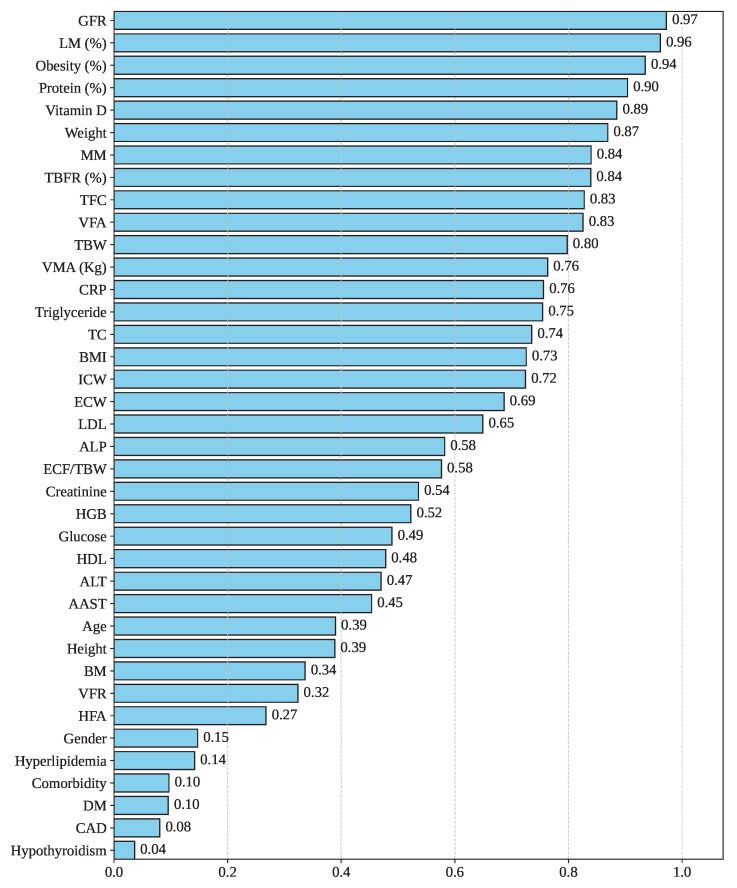
Feature correlation with gallstone status.

Figure 4 illustrates the Cramer’s V correlation between Gallstone Status and the other features in the dataset. Cramer’s V is a statistical measure used to assess the strength of association between variables, particularly suitable for contingency tables larger than 2×2 [22]. As depicted in the figure, the feature GFR exhibits the highest correlation with Gallstone Status, with a score of 0.97. Following that, the features LM, Obesity, Protein, and Vitamin demonstrate strong associations, with Cramer’s V values of 0.96, 0.94, 0.90, and 0.89, respectively. However, Hyperlipidemia, Comorbidity, DM, CAD, and Hypothyroidism have the lowest correlation score of 0.14, 0.10, 0.10, 0.08, and 0.04. So, GFR, LM, and Obesity have a greater impact on gallstone formation, whereas DM, CAD, and Hypothyroidism have a lesser influence on gallstone formation.

### 2.2. Random Forest Classifier

Random Forest (RF) classifier, introduced by Breiman [23], is an ensemble-based machine learning method that constructs multiple decision trees using randomly sampled subsets of the dataset and features. This approach enhances predictive accuracy and mitigates overfitting. RF leverages bootstrap aggregation (bagging) along with random feature selection during tree construction [24]. The ensemble of base learners (decision trees) can be represented using Equation (1) [23].

(1) $$\left\{g\left(x,\Phi_{i}\right)\right\}$$

Here, i=1,2,3,…, and $\Phi_{i}$ represents a randomly drawn parameter vector that guides the learning process for each base learner, g(·), identifying the class for the input vector *x*. The performance of each decision tree is evaluated using the Gini impurity, which is defined in Equation (2) [24].

(2) $$\mathrm{Gini}\left(s\right)=1-\sum_{j=1}^{M}P{\left(D_{j}|s\right)}^{2}$$

In this expression, *s* denotes a split or node in the tree, *M* is the total number of classes, and $D_{j}$ refers to the $j^{\mathrm{th}}$ class label. The majority voting among all individual trees determines the final output. The generalization error of an RF classifier is denoted as $E_{\mathrm{gen}}$ shown in Equation (3) and is defined by the probability that the margin function m(X,Z) is less than zero over the joint distribution of the input vector *X* and its true label *Z*:

(3) $$E_{\mathrm{gen}}=P_{X,Z}\left(m\left(X,Z\right)<0\right)$$

RF is widely recognized for its high predictive accuracy, robustness against outliers, scalability to high-dimensional data, interpretability, and computational efficiency in parallel processing environments [25].

### 2.3. Sand Cat Swarm Optimization

Sand Cat Swarm Optimization (SCSO) is a nature-inspired metaheuristic algorithm developed by Seyyedabbasi and Kiani in 2022, which simulates the unique hunting behavior of sand cats in arid desert regions [26]. Sand cats possess a highly evolved ability to detect underground prey using low-frequency hearing. Upon identifying the location of prey, they perform a strategic and rapid leap from an optimal angle to capture it. This natural hunting strategy has been abstracted into two main algorithmic phases: Exploration and Exploitation.

The switching between exploration and exploitation is controlled by the value of *R*, as shown in Equation (4), where rand is a random number between 1 and 0 and $overliner_{G}$ is varied from 2 to 0 and calculated by the Equation (5) [26]. $S_{M}=2$ is the hearing characteristic of the sand cat, $T_{c}$ is the current iteration, and $T_{max}$ is the maximum iteration number. When |R|≤1, exploitation occurs; |R|>1 triggers exploration.

(4) $$R=2\times \bar{I_{sen}}\times rand-\bar{I_{sen}}$$

(5) $$\bar{I_{sen}}=S_{M}-\left(\frac{2\times S_{M}\times T_{c}}{T_{max}+T_{max}}\right)$$

The individual sensitivity of the sand cat is calculated by Equation (6), which assigns unique search radii to each sand cat by scaling $\bar{I_{sen}}$ with randomness [26]. Further, the position in the exploration phase is updated using Equation (7). The agents move relative to the best candidate position (${\mathrm{Loc}}_{bc}$) and their current position (${\mathrm{Loc}}_{c}$), with $\bar{r}$ scaling step size and rand ensuring comprehensive space coverage.

(6) $$\bar{r}=\bar{I_{sen}}\times rand\left(0,1\right)$$

(7) $$\mathrm{Loc}\left(t+1\right)=\bar{r}\cdot \left({\mathrm{Loc}}_{bc}\left(t\right)-rand\left(0,1\right)\cdot {\mathrm{Loc}}_{c}\left(t\right)\right)$$

In the exploitation phase, the sand cats update their position by the value of Loc(t+1) in Equation (8) [26]. It manages prey-attack behavior (|R|≤1). ${\mathrm{Loc}}_{\mathrm{rnd}}$ computes movement direction toward global best (${\mathrm{Loc}}_{b}$), while cos(θ) (with $\theta \in [0^{\circ },360^{\circ }]$ chosen via Roulette Wheel) directs angled pounces.

(8) $$\begin{array}{cc} {\mathrm{Loc}}_{\mathrm{rnd}} & =\left|rand\left(0,1\right)\cdot {\mathrm{Loc}}_{b}\left(t\right)-{\mathrm{Loc}}_{c}\left(t\right)\right|\\ \mathrm{Loc}(t+1) & ={\mathrm{Loc}}_{b}\left(t\right)-\bar{r}\cdot {\mathrm{Loc}}_{\mathrm{rnd}}\cdot \cos\left(\theta \right) \end{array}$$

#### 2.3.1. Justification of Choosing SCSO

The Sand Cat Swarm Optimization (SCSO) algorithm has been selected due to its proven effectiveness in balancing exploration and exploitation during optimization [26]. Extensive benchmarking on 30 test functions, including both classical and complex CEC2019 functions, has shown that SCSO has achieved the best solution in 63.3% of cases, demonstrating superior convergence and robustness compared to other metaheuristic algorithms. Furthermore, SCSO has been successfully applied to various complex engineering design problems, confirming its ability to locate local and global optima reliably. SCSO is effective because it is stable, flexible, simple to implement, derivative-free, and computationally efficient, making it adaptable to a wide range of problems [27]. Previous studies have shown the multidimensional implementation of the SCSO algorithm, such as power management [28], battery quality management [29], biomedical [15], and cancer classifications [16]. These characteristics make SCSO particularly suitable for feature selection and hyperparameter optimization in the gallstone classification task, where efficient search and convergence are critical for reducing computational cost while maintaining high model performance.

#### 2.3.2. Optimizer Problem Development

We have designed our framework in which the dataset has been transformed into a 10-fold cross-validation set, maintaining 70% of the data for training and 30% for testing. Then, each of the train fold sets has been sent for optimization separately. The optimization window has two main tasks: (I) feature reduction and (II) hyperparameter tuning. We have integrated SCSO with the RF classifier to calculate the fitness of each searching agent (cat) participating in the hunting process. F-score has been considered as the cost function. We have used 100 iterations (epochs) and 50 population size. After each iteration, the RF classifier has calculated the cost function for each agent and assigned it as the current best agent. Further, after 100 epochs, the SCSO has returned the global best fitness value, including the best hyperparameter sets and selected features. This whole process has been detailed in Algorithm 1.
**Algorithm 1** MHA-Based Optimization for Each Training Fold Using RF Hyperparameters  1:**Initialize Metaheuristic Optimization**  2:**Step 1: Define Search Space (D)**  3:     Hyperparameter ranges for Random Forest:  4:        $\phi_{1}$: Number of estimators (n_estimators): 100 to 300  5:        $\phi_{2}$: Maximum tree depth (max_depth): 3 to 10  6:        $\phi_{3}$: Minimum samples split (min_samples_split): 2 to 20  7:        $\phi_{4}$: Minimum samples leaf (min_samples_leaf): 1 to 10  8:        $\phi_{5}$: Number of selected features: 1 to 38  9:     Set population size = 50 and number of iterations = 10010:**Step 2: Metaheuristic Search Process**11:**for** g=1 to 100 **do**12:        Select feature subset $X_{g}$13:        Generate parameter set $\phi_{g}=\left\{\phi_{1}^{g},\phi_{2}^{g},\phi_{3}^{g},\phi_{4}^{g}\right\}$14:        Train Random Forest using $X_{g}$ and $\phi_{g}$15:        Compute fitness using F1-score: $\eta_{g}$16:**end for**17:**Step 3: Identify Global Optimum**18:     Find best fitness: $\eta^{*}=\max\left\{\eta_{1},\eta_{2},\ldots ,\eta_{50}\right\}$19:     Extract optimal feature subset $X^{*}$ and parameters $\phi^{*}$20:**End of Optimization**

**Figure 5:**
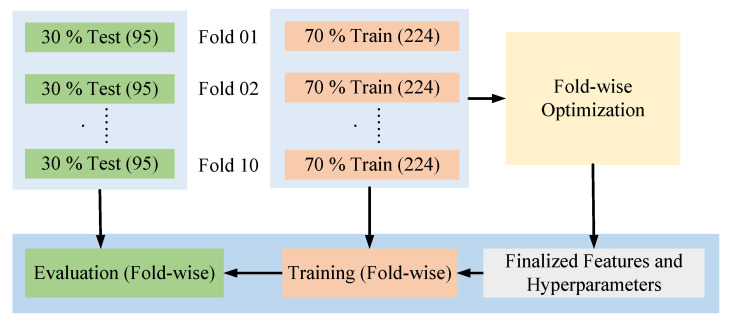
Operational task from optimization to evaluation.

Since each training set in the 10-fold cross-validation has been optimized independently, we obtained 10 different sets of features and hyperparameters. To finalize the model configuration, we applied a majority voting strategy (taking the value of respective parameters that have appeared in the highest number of folds) to determine the optimal hyperparameters and used the union of all selected feature subsets to form the final feature set. Figure 5 presents the main operational view of the process from optimization to evaluation of each distinct fold set, while ensuring no data leakage of the unseen data to the model before the evaluation.

#### 2.3.3. Performance Evaluation

To assess the effectiveness of the developed models, five key evaluation metrics have been utilized: Accuracy, F1-score, Precision, Recall, and Area Under the ROC Curve (AUC). Furthermore, computational efficiency has been examined by analyzing two time-based measures: training time and testing time. The time complexities have been evaluated on a per-fold and per-sample basis. For each fold, we directly measured the total training and testing time. Since each fold contains 224 training samples and 95 testing samples, the average time per sample was obtained by dividing the total time by the corresponding number of samples in each category. Finally, we have presented the average per-sample training and testing time across all folds. The formal definitions of the employed metrics are presented below:

(9) $$\mathrm{Accuracy}=\frac{T_{p}\times T_{n}}{T_{p}+T_{n}+F_{p}+F_{n}}$$

(10) $$\mathrm{Precision}=\frac{T_{p}}{T_{p}+F_{p}}$$

(11) $$\mathrm{Recall}=\frac{T_{p}}{T_{p}+F_{n}}$$

(12) $$F1\text{-}\mathrm{score}=\frac{2\times \mathrm{Precision}\times \mathrm{Recall}}{\mathrm{Precision}+\mathrm{Recall}}$$

(13) $$\mathrm{Training}\text{\_}\mathrm{Time}\text{\_}\mathrm{Per}\text{\_}\mathrm{Sample}=\frac{\mathrm{Time}\text{\_}\mathrm{Per}\text{\_}\mathrm{Fold}}{\mathrm{Training}\;\mathrm{Sample}\;\mathrm{Size}\;(224)}$$

(14) $$\mathrm{Testing}\text{\_}\mathrm{Time}\text{\_}\mathrm{Per}\text{\_}\mathrm{Sample}=\frac{\mathrm{Time}\text{\_}\mathrm{Per}\text{\_}\mathrm{Fold}}{\mathrm{Testing}\;\mathrm{Sample}\;\mathrm{Size}\;(95)}$$

In these formulas, $T_{p}$ (True Positives) refers to the number of instances where the model correctly identifies the positive class (e.g., patients with heart gallstones), while $T_{n}$ (True Negatives) indicates the correct prediction of negative class instances. $F_{p}$ (False Positives) refers to cases where the model incorrectly predicts a negative instance as positive, and $F_{n}$ (False Negatives) captures the positive instances that were misclassified as negative.

#### 2.3.4. SHAP-Based Interpretability

SHapley Additive exPlanations (SHAP) is a popular interpretability framework proposed by Lundberg et al. [30], which builds on the concept of Shapley values from cooperative game theory, originally developed by Lloyd Shapley [31]. SHAP calculates the individual contribution $\varphi_{j}$ of each feature *j* to a particular prediction, as defined by the following equation:

(15) $$\varphi_{j}=\sum_{R\subseteq G\setminus \{j\}}\frac{|R|!\cdot (|G|-|R|-1)!}{|G|!}\left[h_{R\cup \{j\}}\left(x_{R\cup \{j\}}\right)-h_{R}\left(x_{R}\right)\right]$$

Here, *G* represents the complete set of input features, and *R* is any subset of *G* that does not include the feature *j*. The term $h_{R}\left(x_{R}\right)$ denotes the model’s output when only the features in subset *R* are considered, while $h_{R\cup \{j\}}\left(x_{R\cup \{j\}}\right)$ reflects the prediction with feature *j* included.

The final prediction for a given instance *x* can be approximated by summing the expected model output and the SHAP values of all features:

(16) $$h\left(x\right)=E\left[h\left(x\right)\right]+\sum_{j=1}^{m}\varphi_{j}$$

If the value of h(x) exceeds the classification threshold (e.g., 0.5), the instance is classified as Class 1 (e.g., Dengue Positive); otherwise, it is assigned to Class 0.

#### 2.3.5. Diverse Counterfactual Explanations (DiCE)

Diverse Counterfactual Explanations (DiCE) is a model-agnostic technique that generates a set of diverse and plausible counterfactual instances to help users understand how modifications to input features can lead to different model predictions [32]. The DiCE optimization objective is formulated as follows in Equation (17), where C(x) denotes the set of *k* counterfactuals generated for a given input *x*:

(17) $$C\left(x\right)=\arg\min_{c_{1},\ldots ,c_{k}}\frac{1}{k}\sum_{i=1}^{k}\mathrm{yloss}\left(f\left(c_{i}\right),y\right)+\frac{\lambda_{1}}{k}\sum_{i=1}^{k}\mathrm{dist}\left(c_{i},x\right)-\lambda_{2}\cdot \mathrm{dpp}\_\mathrm{diversity}\left(c_{1},\ldots ,c_{k}\right)$$

In this formulation, the term $\mathrm{yloss}(f\left(c_{i}\right),y)$ denotes the classification loss, driving each counterfactual $c_{i}$ toward the specified target class *y*. The proximity component $\mathrm{dist}(c_{i},x)$ discourages counterfactuals that stray too far from the original instance *x*, thereby maintaining realism. Finally, $\mathrm{dpp}\_\mathrm{diversity}(c_{1},\ldots ,c_{k})$ leverages Determinantal Point Processes (DPPs) to encourage variety among the generated counterfactuals, ensuring the returned set offers multiple distinct and actionable alternatives. The hyperparameters $\lambda_{1}$ and $\lambda_{2}$ control the trade-off between proximity and diversity. By optimizing this objective, DiCE provides a diverse set of actionable alternatives that can alter the model’s output, enabling users to explore multiple pathways to achieve a desired prediction, without assuming a single optimal feature modification [32].

## 3. Results

The Results section is structured to assess the performance of classifiers under multiple configurations, facilitating a comprehensive comparison. Initially, evaluations are conducted using classifiers with their default parameters, both without cross-validation and with cross-validation, to observe the baseline performance. Subsequently, the classifiers are re-evaluated after optimization, again considering both scenarios, with and without cross-validation. This layered approach enables a clear understanding of the impact of both parameter tuning and cross-validation on overall classification performance.

### 3.1. Only Classifier Without Cross-Validation

**Figure 6:**
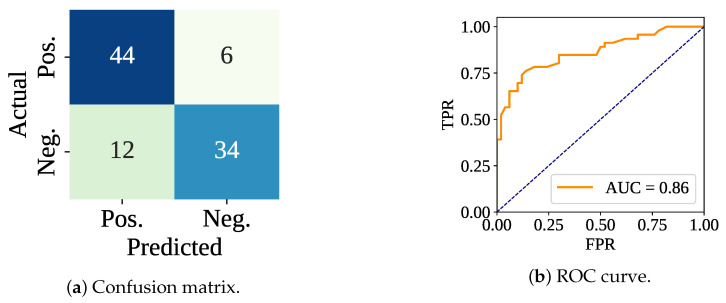
Confusion matrix and ROC curve using only the classifier without cross-validation.

Table 2 shows both the training and testing performances, including the execution time. RF classifier has gained a 100% training accuracy with the default parameters and without cross-validation. However, the test performances have varied. The testing accuracy, F-score, Precision, and Recall have been recorded as 81.25%, 79.07%, 85.00%, and 73.91%, respectively. Figure 6 shows the confusion matrix and ROC curve, in which the model has made 78 (44 $T_{p}$ and 34 $T_{n}$) correct predictions out of 96. In addition, the model has made 6 $F_{n}$ and 12 $F_{P}$ predictions. The ROC curve shows that this time the model has achieved an AUC value of 0.86. The total training samples have taken 230.46 ms to be trained, meaning 1.03 ms per sample. However, the per-sample testing time has been calculated as 0.16 ms.

### 3.2. Only Classifier with Cross-Validation

We have also performed a 10-fold cross-validation operation, using only the classifier. During this operation, we have kept the classifier with default parameters. Table 3 presents the results of a 10-fold cross-validation using only the Random Forest (RF) classifier with its default parameters. For each fold, it has reported the performance metrics—Accuracy, F1-score, Precision, Recall, and computation time—for both the training and testing phases. The classifier has consistently achieved perfect scores (100%) across all training folds, indicating that it has perfectly fitted the training data. However, the test performance has varied across folds, with accuracy ranging from 74.74% to 84.21%. On average, the RF classifier has achieved 78.42% accuracy, 77.75% F1-score, 80.01% precision, and 75.75% recall on the test sets, while maintaining a mean fold-wise training time of 252.93 ms and testing time of 9.82 ms. These results have demonstrated the classifier’s stable generalization capability and computational efficiency.

**Figure 7:**
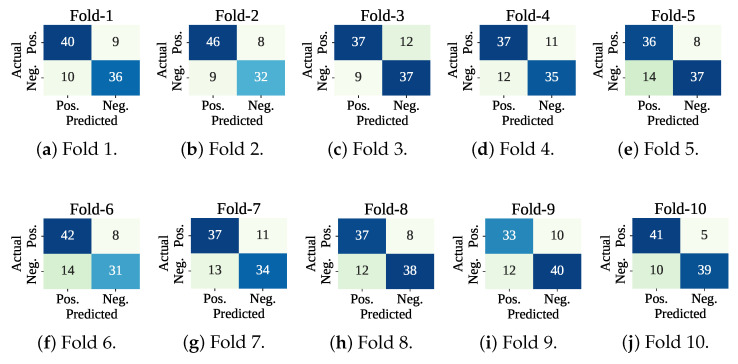
Confusion matrices using only the classifier with cross-validation.

**Figure 8:**
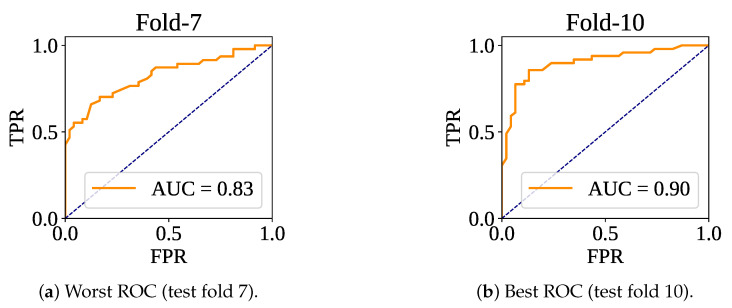
ROC curve for worst and best test fold.

Figure 7 shows the confusion matrices of the folds. The lowest number of misclassifications has been recorded at fold 10, and the highest number of wrong predictions has been found at fold 7. At test fold 10, the model has made 15 wrong predictions ($T_{p}$: 10 and $T_{n}$: 5). At test fold 7, the classifier model has found 24 wrong predictions ($T_{p}$: 13 and $T_{n}$: 11). Figure 8 shows the ROC curve for the fold set 7 (worst AUC: 0.83) and fold set 10 (best AUC: 0.90).

#### 3.2.1. Optimized Classifier with Cross-Validation

**Figure 9:**
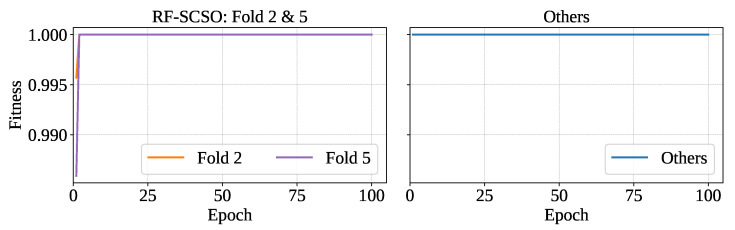
Fitness vs epoch for optimized model.

The RF classifier optimized with SCSO (RF-SCSO) using the training set of each fold separately has minimized the number of features and ensured proper hyperparameter tuning. RF-SCSO has achieved the highest fitness in each fold of optimization. However, training folds 2 and 5 have shown slower convergence in reaching the fitness saturation of 1. The optimizer has achieved fitness of 1 at the first epoch for all training sets except folds 2 and 5, which have reached it at epoch 2. Figure 9 shows the fitness vs. epoch plot for the optimized model.

During the optimization process, each cross-validation fold has independently selected a distinct subset of features along with a corresponding set of hyperparameters. Table 4 presents the optimized feature subsets and hyperparameters obtained from each of the ten folds, including the finalized features and hyperparameters. To construct a robust and generalized model, the finalized feature set has been derived by taking the union of all features selected across the folds, thereby preserving any feature that was deemed important in at least one fold. In contrast, the finalized hyperparameters have been determined using majority voting, where the most frequently occurring values among the folds have been selected as the final configuration.

The table provides a fold-wise breakdown of the selected feature indices and the optimized values of four hyperparameters, denoted as $\phi_{1}$ through $\phi_{4}$. These hyperparameters may correspond to model-specific tuning variables such as the number of estimators, tree depth, or minimum sample split values, depending on the classifier used. Notably, fold-wise variations in both feature selection and hyperparameter values highlight the model’s sensitivity to different data partitions. The final row of the table consolidates the outcome of this optimization strategy: a unified feature set comprising 13 distinct features {6, 7, 8, 12, 16, 20, 25, 27, 30, 32, 33, 35, 37}, and a consistent set of hyperparameters ($\phi_{1}=290$, $\phi_{2}=10$, $\phi_{3}=2$, $\phi_{4}=1$) chosen through statistical mode. This comprehensive strategy ensures that the final model benefits from the diverse insights captured across all folds while maintaining parameter stability for reproducibility and performance.

**Figure 10:**
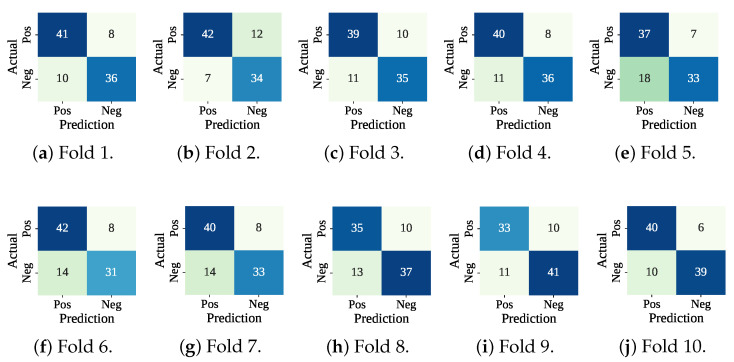
Confusion matrices using RF-SCSO with cross-validation.

**Figure 11:**
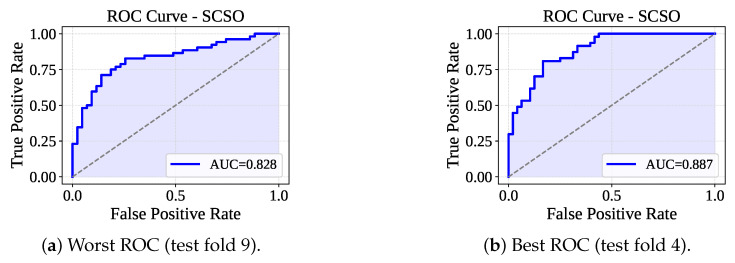
ROC curve for worst and best test fold.

Table 5 presents the performance of the RF-SCSO model evaluated through 10-fold cross-validation. The model has consistently achieved perfect training performance across all folds, with 100% accuracy, F1-score, precision, and recall. On the test sets, the model has shown stable and competitive performance, with an average accuracy of 78.32%, F1-score of 77.44%, precision of 80.36%, and recall of 75.01%. The average fold-wise training and testing times were 736.09 ms and 16.95 ms, respectively. Figure 10 lists the confusion matrices received from the RF-SCSO. Test fold set 5 has faced the maximum misclassification ($F_{p}$: 18 and $F_{n}$: 7), and test fold set 10 has faced the minimum number of misclassifications ($F_{p}$: 10 and $F_{n}$: 6). Figure 11 presents the ROC curve for the test fold set 9 (Worst AUC) and 4 (Best AUC). RF-SCSO has calculated the worst AUC = 0.828 and best AUC = 0.887 for the test folds 9 and 4, respectively.

For balanced comparison of the models, we have additionally performed the RF-SCSO without CV. Table 6 presents the performances of the RF-SCSO without CV, including the total training and testing time for 224 and 95 samples, respectively. The model achieved 100% accuracy, F1-score, precision, and recall on the training set with a training time of 518.94 ms, while on the test set it obtained 79.17% accuracy, 77.78% F1-score, 79.55% precision, and 76.09% recall with a testing time of 21.80 ms. The per-sample training and testing times are 2.32 ms and 0.23 ms, respectively.

#### 3.2.2. Controlling the Overfitting

As there is a notable gap between the training and test accuracy, we have conducted an additional experiment to address the overfitting issue in the only RF classifier without CV, which has demonstrated the highest performance. Specifically, we have trained the RF classifier without CV for different values of the maximum depth, ranging from 1 to 20, to observe its effect on model generalization.

**Figure 12:**
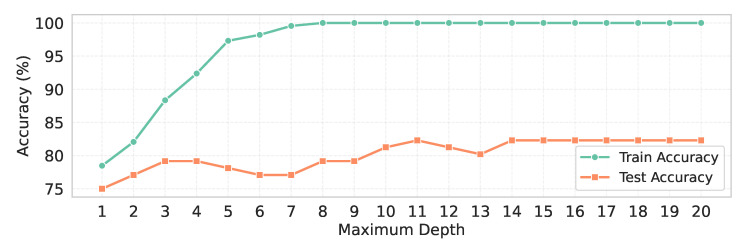
Maximum depth vs. training and test accuracy.

From Table 7, when the maximum depth has been set to lower values (e.g., depth = 1–3), the training accuracy has remained below 75%, and the test accuracy has been below 72%, with a very small gap of around 3%, indicating the absence of overfitting. However, as the maximum depth has increased, the training accuracy has gradually risen, reaching above 95% at higher depths, while the test accuracy has improved only marginally, peaking around 78%. Consequently, a substantial gap between training and test accuracy has emerged, indicating clear overfitting. A matter of consideration is that after the maximum depth has reached 14, both training and test accuracies have become saturated, with the gap stabilizing at approximately 17.71%, as shown in Figure 12.

#### 3.2.3. Computational Complexity

The Table 8 presents the computational time complexity of two models using 10-fold cross-validation. For the standard RF classifier, the fold-wise training time has ranged from 206.77 ms to 499.75 ms, and the training per sample has varied from 0.923 ms to 2.231 ms, with averages of 252.93 ms and 1.129 ms, respectively. The fold-wise testing time has ranged from 8.15 ms to 13.25 ms, and the testing per sample has varied from 0.086 ms to 0.139 ms, with averages of 9.82 ms and 0.103 ms, respectively.

For the RF–SCSO model, the fold-wise training time has ranged from 506.59 ms to 1358.37 ms, and the training per sample has varied from 2.262 ms to 6.064 ms, with averages of 736.09 ms and 3.286 ms, respectively. The fold-wise testing time has ranged from 7.71 ms to 64.64 ms, and the testing per sample has varied from 0.081 ms to 0.680 ms, with averages of 16.95 ms and 0.178 ms, respectively.

#### 3.2.4. Misclassifications

In this subsection, misclassification indices have been presented. We have enlisted the results from the three different approaches to classify the presence of gallstones. We have some similar records that have been misclassified in each approach. Indexes 73, 94, 116, 118, 147, 176, 177, 194, 195, 203, 211, 250, 299, and 316 have been misclassified in each approach (only classifier with and without cross-validation and RF-SCSO with cross-validation). Table 9 and Table 10 have presented the clinical profiles of the misclassified cases, specifically the false negatives (actual gallstone presence predicted as absence) and false positives (gallstone absence predicted as presence). The analysis of the false negative group has revealed that these samples have generally exhibited lower levels of key clinical markers, including CRP (mean = 0.718), Creatinine (mean = 0.734), and AAST scores (mean = 15.4). All patients in this group have had no diabetes (DM = 0), with relatively moderate obesity percentages (mean = 31.74%) and lower Glucose (mean = 94.8) and LDL (mean = 161.2) values. These characteristics have likely contributed to a subtler clinical presentation, which has led the model to misclassify them as gallstone-negative.

Conversely, the false positive group has shown higher values for several metabolic and inflammatory features, such as Glucose (mean = 115.67), ALP (mean = 81.89), and LDL (mean = 123.22), which may have mimicked the profiles of gallstone-positive cases. Some patients in this group have had diabetes (DM = 1 in two cases), and obesity levels have been more variable (mean = 27.64%). Additionally, higher ICW (mean = 24.17) and Weight (mean = 82.71 kg) have been observed, which may have biased the model toward a positive prediction.

### 3.3. SHAP Analysis

In this SHAP analysis, we have presented the mean SHAP plot and summary plot to visualize the feature ranking and contribution to the overall model. Then we have used a waterfall plot for depth analysis of the individual predictions, especially for misclassification.

#### 3.3.1. SHAP of Only Classifier Without Cross-Validation

**Figure 13:**
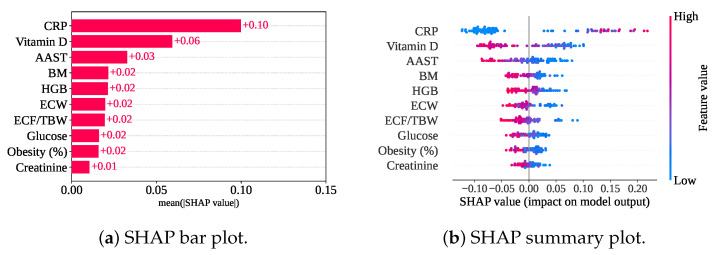
SHAP plots for only the classifier without cross-validation.

Figure 13 shows the SHAP plots for the top ten features based on only the RF classifier without a cross-validation model. SHAP has found that CRP has the highest mean SHAP value (+0.10), indicating the highest contribution to the model. Vitamin D, AAST, BM, and HGB are the next four top contributors to the model, with the mean SHAP value of +0.06, +0.03, +0.02, and +0.02, respectively, as shown in Figure 13b.

Figure 13a shows a summary plot of the only classifier model without cross-validation. A lower value (blue color) of CRP has mainly contributed to the model with a negative SHAP value. The negative SHAP value of the feature has pulled the decision towards class 0 (gallstone positive). However, the higher value (pink color) of other features has contributed to the model’s negative SHAP value, and this has also led to the decision in favor of the gallstone-positive class.

#### 3.3.2. SHAP of Only Classifier with Cross-Validation

**Figure 14:**
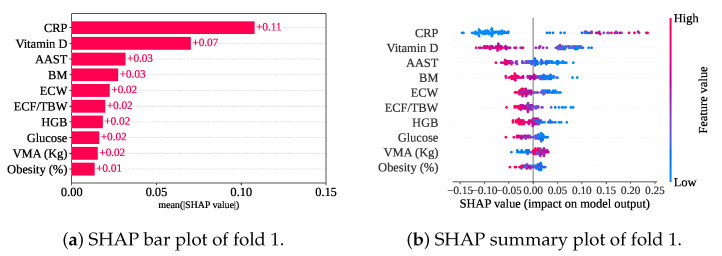
SHAP plots for only the classifier with cross-validation.

Figure 14 shows the measured mean SHAP value of +0.11 (CRP),+0.07 (Vitamin D), +0.03 (AAST and BM), +0.02 (ECW, ECF/TBW, HGB, Glucose, and VMA), and +0.01 (Obesity). So, CRP, Vitamin D, AAST, BM, and ECW are the top five features for this model, too. In comparison between only the classifier with and without cross-validation, HGB has been replaced with ECW from the fifth position. Nevertheless, the summary plot has also shown the feature impact on the model. In cross-validation with only a classifier, the summary plot shows a similar nature. In addition, a lower VMA has achieved a negative SHAP value, leading the prediction towards the gallstone-positive group.

#### 3.3.3. SHAP for RF-SCSO

**Figure 15:**
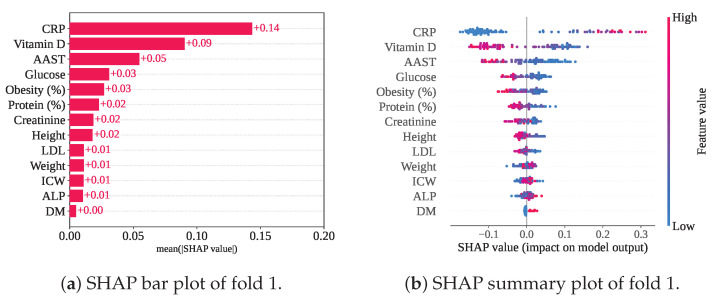
SHAP plots for RF-SCSO with cross-validation.

Figure 15 illustrates the mean SHAP values and summary plot, highlighting the rankings of the selected features within the RF-SCSO framework. Among the 13 selected features, CRP has consistently emerged as the top-ranked contributor, as observed in previous frameworks. Notably, lower CRP levels have been associated with negative SHAP values, indicating a link with Class 0 (gallstone positive). Vitamin D and AAST have followed CRP in importance, with mean SHAP values of +0.09 and +0.05, respectively, while CRP has maintained the highest mean SHAP value of +0.14. Additionally, higher levels of Vitamin D, AAST, Glucose, and Obesity have influenced the model towards classifying individuals as gallstone positive.

**Figure 16:**
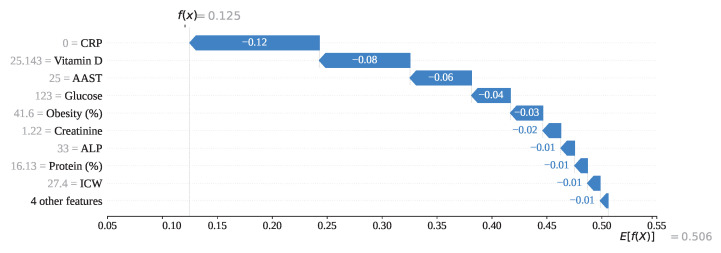
Individual prediction of $T_{p}$ (Index: 25).

To understand the model at a fundamental level, we have conducted an in-depth analysis of individual predictions, covering each type: $T_{p}$ (Index: 25), $T_{n}$ (Index: 208), $F_{p}$ (Index: 176), and $F_{n}$ (Index: 76). Figure 16 illustrates the prediction formation process for index 208 using a SHAP-generated waterfall plot. In this case, CRP, with a feature value of 0, has contributed the most to the prediction with a SHAP value of −0.12. Additionally, features such as Vitamin D (−0.08), AAST (−0.06), Glucose (−0.04), and Obesity (−0.03), along with other contributing features, have all exhibited negative SHAP values. Starting from the base value E[f(x)]=0.506, these cumulative negative contributions have led to a final output of f(x)=0.125, which is below the classification threshold of 0.5. Consequently, the model has correctly predicted the instance as class 0 (gallstone positive).

**Figure 17:**
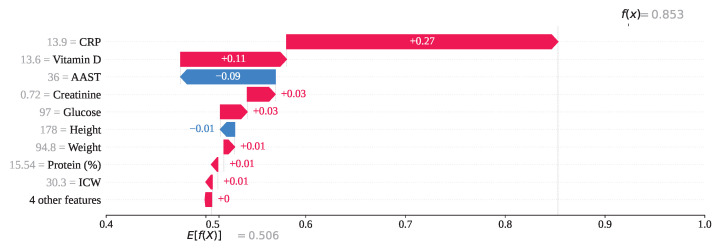
Individual prediction of $T_{n}$ (Index: 208).

For index 208, the feature CRP with a value of 13.9 has exhibited the highest SHAP value of +0.27, indicating a strong positive influence on the model’s output as shown in Figure 17. Vitamin D, with a feature value of 13.6, has also contributed positively with a SHAP value of +0.11. In contrast, AAST, having a value of 36, has contributed negatively with a SHAP value of −0.09. All other features, except Height, have shown a positive influence on the prediction outcome. Consequently, the model has classified this instance as class 1 (gallstone negative), with a final SHAP-based output value of f(x)=0.85, which exceeds the decision threshold of 0.5.

**Figure 18:**
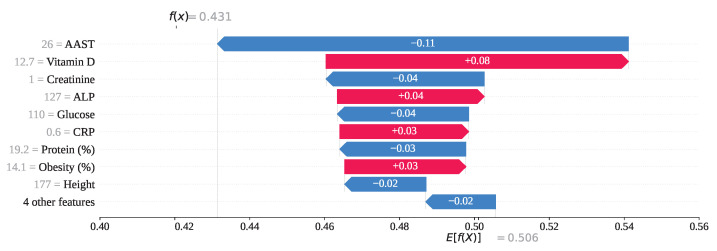
Individual prediction of $F_{p}$ (Index: 176).

For index 176 in Figure 18, the feature AAST, with a value of 26, has ranked first in influence with a SHAP value of −0.11, strongly contributing to the prediction of gallstone positivity. Additionally, features such as Creatinine = 1, Glucose = 110, Protein = 19.2, and Height = 177 have also yielded negative SHAP values, collectively steering the model toward an incorrect prediction of class 0. In contrast, Vitamin D = 12.7, ALP = 127, CRP = 0.6, and Obesity = 14.1 have produced positive SHAP values, attempting to counterbalance the misclassification. Ultimately, the cumulative SHAP value for this instance has been computed as f(x)=0.431, which falls below the 0.5 threshold. Therefore, the model has predicted this individual as gallstone-positive, although the true label is gallstone-negative.

**Figure 19:**
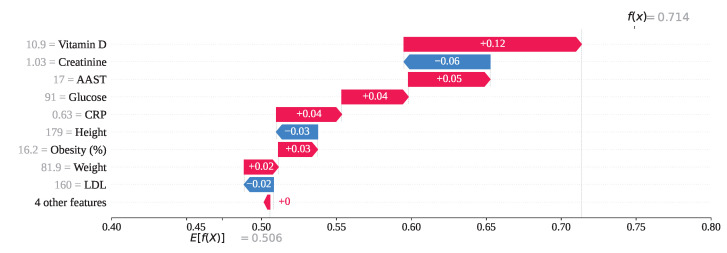
Individual prediction of $F_{n}$ (Index: 73).

For index 73 in Figure 19, several features have positively influenced the model’s prediction. Specifically, Vitamin D, AAST, Glucose, CRP, Obesity, and Weight have contributed SHAP values of +0.12, +0.05, +0.04, +0.03, +0.02, and +0.02, respectively. As a result of these positive contributions, the overall SHAP output value has been calculated as f(x)=0.714, leading the model to predict class 1 (gallstone negative). However, this prediction is incorrect, as the individual has gallstones. On the other hand, Creatinine = 1.03, Height = 179, and LDL = 160 have exhibited negative SHAP values of −0.06, −0.03, and −0.02, respectively, attempting to steer the model toward the correct classification. Despite their influence, these features were insufficient to override the stronger positive contributions, resulting in a misclassification.

### 3.4. DiCE Analysis

In all of the developed frameworks, CRP, Vitamin D, and AAST have consistently emerged as the top three most influential features based on feature selection and SHAP analyses. To further understand their contribution to individual predictions, we have conducted a DiCE analysis focused specifically on these features. Table 11 illustrates ten counterfactual scenarios generated for the subject at index 176, who in reality belongs to the non-gallstone class (class 1). However, the model has incorrectly classified this individual as having gallstones (class 0), making it a false positive prediction. The goal of this counterfactual analysis is to determine how minimal, feasible changes to the input features could reverse the model’s incorrect decision and result in a correct classification.

As shown in the table, the most frequent and impactful change observed across all ten counterfactuals is the adjustment of CRP levels. In every counterfactual instance, the original CRP value of 0.6 has been increased, with values ranging from 3.0 to 15.2, which alone or in combination with slight adjustments in Vitamin D or AAST, successfully flip the model’s prediction from class 0 to class 1. For example, in CF#1, simply increasing CRP from 0.6 to 12.8 is sufficient to achieve a correct prediction, while in CF#2, a simultaneous increase in both CRP (0.6 → 14.7) and AAST (26.0 → 144.9) is necessary. Similarly, in CF#3 and CF#4, the prediction is corrected by moderate increases in both CRP and Vitamin D. Interestingly, CRP is the only feature that is altered in all ten counterfactuals, which strongly suggests that CRP is the most decisive feature influencing the model’s classification for this individual. Changes in Vitamin D and AAST occur less frequently and are often secondary, indicating their supportive but less dominant roles.

We have conducted a DiCE-based counterfactual analysis for the individual at index 76, whose actual label is gallstone positive (class 0). However, the models have incorrectly classified this case as gallstone negative (class 1), resulting in a false prediction. Table 12 presents the ten counterfactual instances for this case. Notably, in all counterfactuals, the CRP value remains unchanged at 0.63, suggesting that CRP has had minimal influence on this particular decision. Instead, the model’s prediction has been altered primarily through increases in Vitamin D and AAST values. In the majority of counterfactuals, either Vitamin D or AAST—or both—have been significantly increased. For example, in CF#1, Vitamin D has been adjusted from 10.9 to 18.1 and AAST from 17.0 to 60.4, which successfully corrects the model’s prediction. Similarly, CF#2 and CF#3 show a strong upward shift in both Vitamin D (to over 36.0) and AAST (to over 120.0), effectively flipping the model output. In some cases (e.g., CF#5), only Vitamin D has been changed (from 10.9 to 39.7), whereas in others like CF#6 and CF#9, only AAST has been modified, indicating that either feature alone can sometimes suffice to reverse the prediction.

This pattern suggests that, for index 76, the model has placed significantly more weight on AAST and Vitamin D than on CRP in its decision-making process. The absence of any change in CRP across all ten counterfactuals reinforces this observation. Therefore, we can infer that misclassification in this case can be corrected by appropriate adjustments to Vitamin D and AAST values, highlighting their dominant influence in the prediction for this specific individual. Overall, the counterfactual explanations not only help correct a false negative prediction but also offer actionable insights into the specific features that govern the model’s decisions at an individual level.

To strengthen the DiCE explanation, we have incorporated the alteration of a correct prediction for index 208, a gallstone-positive individual correctly classified by the RF-SCSO model. We have generated five counterfactuals for this index to explore how the prediction could change from positive to negative. Table 13 presents these five counterfactuals. The analysis has demonstrated how altering key feature values can modify the model’s prediction. Specifically, lowering the CRP value from 13.9 to values between 0.1 and 0.5 has been sufficient to change the prediction from gallstone-positive (1) to gallstone-negative (0), while keeping Vitamin D and AAST largely unchanged. In the fifth counterfactual, simultaneous changes in CRP and Vitamin D have further illustrated how combinations of feature alterations have influenced the prediction. These results have highlighted the sensitivity of the RF-SCSO model to CRP and Vitamin D, and have demonstrated the practical utility of DiCE in identifying actionable feature-level interventions to correct misclassifications.

## 4. Discussion

In this study, we have implemented three machine learning (ML) approaches to classify individuals as gallstone-positive or gallstone-negative using a tabular dataset. While numerous ML-based models exist for gallstone detection, the majority rely on image-based data. Table 14 presents a comparative analysis between our models and existing approaches. According to the literature [5,6,7,8], image-based classification models have achieved accuracies of up to 98%. In contrast, Esen et al. employed a tabular dataset and reported an accuracy of 85.42%, albeit using a substantially large feature set comprising 38 variables.

In comparison, our proposed framework using only a Random Forest classifier without cross-validation (RF without CV) has achieved an accuracy of 81.25%, an F-score of 79.07%, a precision of 85%, and a recall of 73.91% using all 38 features. These results demonstrate strong compatibility with existing gallstone prediction models. To enhance the generalizability of the model, we have further employed a 10-fold cross-validation approach (RF with CV), which has yielded a mean accuracy of 78.42%, an F-score of 77.75%, a precision of 80.01%, and a recall of 75.75% using the same 38 features. Subsequently, we have applied the SCSO algorithm to reduce the number of input features while maintaining competitive performance. RF-SCSO without CV has achieved 79.17% accuracy, 77.78% F-score, 79.55% precision, and 76.09% recall, using 13 features. The final framework, RF-SCSO with CV, has achieved a mean accuracy of 78.32%, an F-score of 77.44%, a precision of 80.36%, and a recall of 75.01% using only 13 features. These findings indicate that the reduced feature set yields performance comparable to the full-feature RF with CV model, highlighting the effectiveness of the proposed optimization-based feature selection.

Moreover, this research has identified CRP, Vitamin D, and AAST as the most influential features for gallstone prediction. CRP is associated with gallstone-related inflammation and disease severity, serving as a useful biomarker in gallbladder disorders [33]. Another medical research on 4484 participants has found that CRP levels are associated with a greater prevalence of gallstones [34]. Liu et al. have claimed that CRP is an independent risk factor for gallstone disease [35]. Another study on 6873 people has shown that higher dietary vitamin D consumption (D2+D3) is positively associated with gallstone incidence [36]. AAST is a liver enzyme; a higher level of AAST has a positive association with gallstone formation [37]. Another study on 55,531 has shown that liver enzymes may increase the risk of developing gallstones [38]. So, previous studies have proven the association of CRP, Vitamin D, and AAST with gallstone classification. Our study has also identified these three features as the top three important features.

Variations in the values of CRP, Vitamin D, and AAST can significantly impact the model’s output and may even lead to misclassification, as demonstrated in the analysis of index 176 and index 73. For index 176, a $F_{p}$ prediction is incorrect, which has AAST = 26, Vitamin D = 12.7, and CRP = 0.6. In this case, AAST = 26 has played a vital role in making this wrong prediction. For index 76, an $F_{n}$ prediction is incorrect, in which Vitamin D (10.9), AAST (17), and CRP (0.63) have pushed the prediction to be class 1 (gallstone negative). Table 15 shows the mean value of the top three features separately for class 0 and class 1. In the original dataset, the mean feature values for a person with a gallstone are CPR = 0.46, Vitamin D = 24.90, and AAST = 23.91. Further, these values for the gallstone negatives are 3.27, 17.83, and 19.41, respectively, for CRP, Vitamin D, and AAST. The CRP value in the gallstone-positive group is lower than in the negative group. However, Vitamin D and AAST have higher levels in the gallstone-positive group compared to the gallstone-negative group. For the wrong prediction (predicted as class 1 or gallstone negative) indices shown in Table 9, the mean values of the selected three features are 0.718, 16.626, and 15.4, respectively, for CRP, Vitamin D, and AAST.

On the other side, for the wrong prediction (predicted as class 0 or gallstone positive) indices shown in Table 10, the mean values of the selected three features are 0.279, 23.31, and 21.00, respectively, for CRP, Vitamin D, and AAST. This has shown a clear difference between the mean feature values of the wrong predicted indices and has a relation with the mean of the original dataset.

In the dataset, the gallstone-positive individuals have a mean CRP of 0.46, which is lower than the negative individuals. Further, the $F_{p}$ indices also have a mean CRP of 0.279, which is surprisingly low. Not only that, in the original dataset, the positive cases have a higher mean of Vitamin D and AAST, as shown in the $F_{p}$ cases. Because of these similarities in feature values, the models have classified them as $F_{p}$. The same relation we have also identified for $F_{n}$ cases with the feature mean value of the negative individuals in the original dataset. So this research has shown that the individuals who have lower CRP, higher Vitamin D, and AAST, have a higher chance of gallstones. DiCE has also mentioned the same relation as shown in Table 11 and Table 12.

Additionally, we have presented that the overfitting issue can be solved by reducing the maximum depth. At lower depths, the accuracy has been relatively low, but the gap between training and test performance has been minimal, while higher depths have provided better accuracy but have raised the concern of overfitting. So, the main contribution of this paper relies on the reduction of features, resolving the over-fitting issue, and enhancing model interpretability, supported by SHAP-based feature importance analysis and DiCE-driven exploration of misclassifications and error correction.

In clinical practice, the findings from SHAP and DiCE can play an essential role. As SHAP has identified the most influential features (CRP, Vitamin D, AAST), clinicians can place greater emphasis on these features during diagnosis. Additionally, SHAP has demonstrated how the model generates individual predictions, allowing clinicians to determine which features are playing a key role for a particular patient. The clinical computational efficiency has been achieved by reducing the original 38 features to 13 optimized features, thereby minimizing diagnostic tests, lowering computational burden, and enabling faster clinical decision-making. Finally, the DiCE explanations can assist in addressing misclassifications and anomalies in diagnosis, providing actionable insights for improving patient assessment.

Future Scope: This study has successfully classified gallstone presence using a tabular dataset and has shown commendable performance with the use of RF and SCSO. Beyond achieving reliable prediction, it has also provided important insights into feature rankings, causes of misclassification, and ways to correct incorrect predictions. These outcomes have the potential to contribute significantly to the medical field. In the future, this framework can be integrated into advanced medical technologies such as smart diagnosis systems, autonomous disease detection tools, and smartphone-based health monitoring applications. Such integration may enhance early detection, support clinical decision-making, and improve overall patient care.

Limitations: This study has several limitations that should be acknowledged. First, the dataset is relatively small (319 subjects), which restricts the statistical power and robustness of the findings. Second, as the data were collected from a single center, the results may be limited in their generalizability to broader populations with diverse demographic and clinical characteristics. Third, the absence of an external dataset with the same feature set prevented independent validation, which is critical for confirming the robustness and clinical applicability of our models. Finally, we observed a discrepancy between training and testing performance, suggesting a potential risk of overfitting, possibly driven by the limited sample size. Future work should focus on incorporating larger, multi-center datasets with external validation to improve the generalizability and reliability of gallstone prediction models.

## 5. Conclusions

Gallstones are hard crystalline formations in the gallbladder or bile ducts, primarily caused by excess cholesterol or bilirubin in bile, and affect approximately 10–20% of adults worldwide. Recent research has demonstrated the growing acceptance of ML-based approaches for gallstone prediction using both image and tabular data. In this study, we have proposed a Random Forest (RF) classifier optimized with the Sand Cat Swarm Optimization (SCSO) algorithm. Our only RF without a CV framework has achieved an accuracy of 81.25%, an F-score of 79.07%, a precision of 85%, and a recall of 73.91% using 38 features, while RF with CV has obtained a 10-fold cross-validation mean accuracy of 78.42% with the same feature set. Furthermore, the optimized RF-SCSO without and with CV framework has achieved accuracy of 79.17% and 78.32% (mean accuracy), respectively, using only 13 features, indicating that SCSO has provided significant feature reduction with minimal performance loss. To enhance interpretability, SHAP has been employed to explain the model’s predictions, revealing that CRP, Vitamin D, and AAST are the three most influential features across all frameworks. Additionally, DiCE has demonstrated the error correction mechanism for False Positive ($F_{p}$) and False Negative ($F_{n}$) predictions. These findings underscore the potential of combining RF and SCSO for efficient and interpretable gallstone prediction.

## Figures and Tables

**Table 1:** Feature overview of the gallstone dataset (Fet. = Feature; Bin. = Binary; Cat. = Categorical; Con. = Continuous; Int.=Integer).

Fet. Index	Feature Name	Type	Description
Target	Gallstone Status	Bin.	Presence (0) or absence (1) of gallstones
0	Age	Int.	Patient’s age
1	Gender	Cat.	Patient’s sex
2	Comorbidity	Cat.	Other existing diseases
3	Coronary Artery Disease (CAD)	Bin.	Heart disease presence
4	Hypothyroidism	Bin.	Underactive thyroid presence
5	Hyperlipidemia	Bin.	Elevated blood fats
6	Diabetes Mellitus (DM)	Bin.	Diabetes presence
7	Height	Int.	Patient’s height
8	Weight	Con.	Patient’s weight
9	Body Mass Index (BMI)	Con.	Weight-to-height ratio
10	Total Body Water (TBW)	Con.	Total body water volume
11	Extracellular Water (ECW)	Con.	Water outside cells
12	Intracellular Water (ICW)	Con.	Water inside cells
13	ECF/TBW Ratio	Con.	Ratio of extracellular fluid to total water
14	Total Body Fat Ratio (TBFR)	Con.	Percentage of body fat
15	Lean Mass (LM)	Con.	Lean tissue mass
16	Body Protein Content (Protein)	Con.	Total protein amount
17	Visceral Fat Rating (VFR)	Int.	Fat around organs
18	Bone Mass (BM)	Con.	Bone tissue mass
19	Muscle Mass (MM)	Con.	Muscle tissue mass
20	Obesity	Con.	Level of excess fat
21	Total Fat Content (TFC)	Con.	Total fat quantity
22	Visceral Fat Area (VFA)	Con.	Area of visceral fat
23	Visceral Muscle Area (VMA)	Con.	Area of visceral muscle
24	Hepatic Fat Accumulation (HFA)	Cat.	Liver fat buildup
25	Glucose	Con.	Blood sugar level
26	Total Cholesterol (TC)	Con.	Total cholesterol level
27	Low Density Lipoprotein (LDL)	Con.	“Bad” cholesterol level
28	High Density Lipoprotein (HDL)	Con.	“Good” cholesterol level
29	Triglyceride	Con.	Blood triglyceride level
30	Aspartat Aminotransferaz (AAST)	Con.	Liver enzyme level
31	Alanin Aminotransferaz (ALT)	Con.	Liver enzyme level
32	Alkaline Phosphatase (ALP)	Con.	Liver and bone enzyme
33	Creatinine	Con.	Kidney function marker
34	Glomerular Filtration Rate (GFR)	Con.	Kidney filtration rate
35	C-Reactive Protein (CRP)	Con.	Inflammation marker
36	Hemoglobin (HGB)	Con.	Oxygen-carrying blood protein
37	Vitamin D	Con.	Vitamin level for bone health

**Table 2:** Performance using only RF classifier without cross-validation (Acc. = Accuracy; F1 = F-score; Pre. = Precision; Rec. = Recall; ms = milliseconds).

Train						Test				
Acc. (%)	F1 (%)	Pre. (%)	Rec. (%)	Time (ms)		Acc. (%)	F1 (%)	Pre. (%)	Rec. (%)	Time (ms)
100.00	100.00	100.00	100.00	230.46		81.25	79.07	85.00	73.91	15.03

**Table 3:** Performance using only RF classifier with 10-fold cross-validation (Acc. = Accuracy; F1 = F-score; Pre. = Precision; Rec. = Recall; ms = milliseconds).

Fold	Train						Test				
	Acc. (%)	F1 (%)	Pre. (%)	Rec. (%)	Time (ms)		Acc. (%)	F1 (%)	Pre. (%)	Rec. (%)	Time (ms)
1	100.00	100.00	100.00	100.00	499.75		80.00	79.12	80.00	78.26	9.22
2	100.00	100.00	100.00	100.00	207.56		82.11	79.01	80.00	78.05	12.63
3	100.00	100.00	100.00	100.00	210.55		77.89	77.89	75.51	80.43	8.50
4	100.00	100.00	100.00	100.00	206.77		75.79	75.27	76.09	74.47	8.74
5	100.00	100.00	100.00	100.00	216.70		76.84	77.08	82.22	72.55	9.08
6	100.00	100.00	100.00	100.00	303.06		76.84	73.81	79.49	68.89	13.25
7	100.00	100.00	100.00	100.00	207.28		74.74	73.91	75.56	72.34	11.16
8	100.00	100.00	100.00	100.00	224.96		78.95	79.17	82.61	76.00	8.15
9	100.00	100.00	100.00	100.00	219.02		76.84	78.43	80.00	76.92	8.50
10	100.00	100.00	100.00	100.00	233.61		84.21	83.87	88.64	79.59	8.97
Mean	100.00	100.00	100.00	100.00	252.93		78.42	77.75	80.01	75.75	9.82

**Table 4:** Fold-wise and finalized optimized hyperparameters and selected features.

Fold	Feature Indexes	$\phi_{1}$	$\phi_{2}$	$\phi_{3}$	$\phi_{4}$
1	30, 33, 37	290	10	2	1
2	16, 30, 33	273	10	2	1
3	27, 33, 35, 37	105	10	2	1
4	16, 25, 33	156	9	2	1
5	6, 7, 8, 20, 27	228	10	2	1
6	12, 33, 35	252	10	2	1
7	16, 37	136	10	2	1
8	25, 30, 37	178	10	2	1
9	7, 27, 30, 32	290	10	2	1
10	8, 16	290	9	2	1
Finalized	6, 7, 8, 12, 16, 20, 25, 27, 30, 32, 33, 35, 37	290	10	2	1

**Table 5:** Performance using RF–SCSO with 10-fold cross-validation (Acc. = Accuracy; F1 = F-score; Pre. = Precision; Rec. = Recall; ms = milliseconds).

Fold	Train						Test				
	Acc. (%)	F1 (%)	Pre. (%)	Rec. (%)	Time (ms)		Acc. (%)	F1 (%)	Pre. (%)	Rec. (%)	Time (ms)
1	100.00	100.00	100.00	100.00	1126.85		81.05	80.00	81.82	78.26	64.64
2	100.00	100.00	100.00	100.00	1358.37		80.00	78.16	73.91	82.93	26.65
3	100.00	100.00	100.00	100.00	794.87		77.89	76.92	77.78	76.09	13.43
4	100.00	100.00	100.00	100.00	523.95		80.00	79.12	81.82	76.60	7.73
5	100.00	100.00	100.00	100.00	506.59		73.68	72.53	82.50	64.71	7.99
6	100.00	100.00	100.00	100.00	529.04		76.84	73.81	79.49	68.89	9.80
7	100.00	100.00	100.00	100.00	812.72		76.84	75.00	80.49	70.21	10.35
8	100.00	100.00	100.00	100.00	517.15		75.79	76.29	78.72	74.00	8.34
9	100.00	100.00	100.00	100.00	677.25		77.89	79.61	80.39	78.85	12.89
10	100.00	100.00	100.00	100.00	514.08		83.16	82.98	86.67	79.59	7.71
Mean	100.00	100.00	100.00	100.00	736.09		78.32	77.44	80.36	75.01	16.95

**Table 6:** Performance using RF-SCSO classifier without cross-validation (Acc. = Accuracy; F1 = F-score; Pre. = Precision; Rec. = Recall; ms = milliseconds).

Train						Test				
Acc. (%)	F1 (%)	Pre. (%)	Rec. (%)	Time (ms)		Acc. (%)	F1 (%)	Pre. (%)	Rec. (%)	Time (ms)
100.00	100.00	100.00	100.00	518.94		79.17	77.78	79.55	76.09	21.80

**Table 7:** Performance of only RF without CV for different maximum depths (Acc. = Accuracy; F1 = F-score; Pre. = Precision; Rec. = Recall; Gap = Train-Test Accuracy Gap).

Max Depth	Train					Test				Gap (%)
	Acc. (%)	F1 (%)	Pre. (%)	Rec. (%)		Acc. (%)	F1 (%)	Pre. (%)	Rec. (%)	
1	78.48	76.70	82.29	71.82		75.00	73.33	78.57	68.75	3.48
2	82.06	81.13	84.31	78.18		77.08	76.09	79.55	72.92	4.98
3	88.34	87.62	92.00	83.64		79.17	78.72	80.43	77.08	9.17
4	92.38	91.87	96.97	87.27		79.17	79.59	78.00	81.25	13.21
5	97.31	97.20	100.00	94.55		78.13	77.89	78.72	77.08	19.18
6	98.21	98.15	100.00	96.36		77.08	77.55	76.00	79.17	21.12
7	99.55	99.54	100.00	99.09		77.08	77.08	77.08	77.08	22.47
8	100.00	100.00	100.00	100.00		79.17	79.59	78.00	81.25	20.83
9	100.00	100.00	100.00	100.00		79.17	79.59	78.00	81.25	20.83
10	100.00	100.00	100.00	100.00		81.25	82.00	78.85	85.42	18.75
11	100.00	100.00	100.00	100.00		82.29	82.83	80.39	85.42	17.71
12	100.00	100.00	100.00	100.00		81.25	82.00	78.85	85.42	18.75
13	100.00	100.00	100.00	100.00		80.21	80.81	78.43	83.33	19.79
14	100.00	100.00	100.00	100.00		82.29	83.17	79.25	87.50	17.71
15	100.00	100.00	100.00	100.00		82.29	83.17	79.25	87.50	17.71
16	100.00	100.00	100.00	100.00		82.29	83.17	79.25	87.50	17.71
17	100.00	100.00	100.00	100.00		82.29	83.17	79.25	87.50	17.71
18	100.00	100.00	100.00	100.00		82.29	83.17	79.25	87.50	17.71
19	100.00	100.00	100.00	100.00		82.29	83.17	79.25	87.50	17.71
20	100.00	100.00	100.00	100.00		82.29	83.17	79.25	87.50	17.71

**Table 8:** Computational time complexity of the models (ms = milliseconds).

Only RF Classifier with 10-fold Cross-Validation					
Fold	Training Time			Testing Time	
	Fold-Wise (ms)	Per Sample (ms)		Fold-Wise (ms)	Per Sample (ms)
01	499.75	2.231		9.22	0.097
02	207.56	0.927		12.63	0.133
03	210.55	0.940		8.50	0.089
04	206.77	0.923		8.74	0.092
05	216.70	0.967		9.08	0.096
06	303.06	1.353		13.25	0.139
07	207.28	0.925		11.16	0.117
08	224.96	1.004		8.15	0.086
09	219.02	0.978		8.50	0.089
10	233.61	1.043		8.97	0.094
Mean	252.93	1.129		9.82	0.103
**RF–SCSO with 10-fold cross-validation**					
**Fold**	**Training Time**			**Testing Time**	
	**Fold-wise (ms)**	**Per Sample (ms)**		**Fold-wise (ms)**	**Per Sample (ms)**
01	1126.85	5.031		64.64	0.680
02	1358.37	6.064		26.65	0.281
03	794.87	3.549		13.43	0.141
04	523.95	2.339		7.73	0.081
05	506.59	2.262		7.99	0.084
06	529.04	2.362		9.80	0.103
07	812.72	3.628		10.35	0.109
08	517.15	2.309		8.34	0.088
09	677.25	3.023		12.89	0.136
10	514.08	2.295		7.71	0.081
Mean	736.09	3.286		16.95	0.178

**Table 9:** False negative predictions.

Feature	Index 73	Index 116	Index 118	Index 147	Index 94	Mean
ALP	66	157	46	70	92	86.2
Creatinine	1.03	0.69	0.64	0.68	0.63	0.734
CRP	0.63	0.18	0.00	2.00	0.78	0.718
Vitamin D	10.9	13.7	8.23	28.5	21.8	16.626
DM	0	0	0	0	0	0.0
Height	179	156	161	146	158	160.0
Weight	81.9	56.9	70.4	63.6	92.5	73.06
ICW	28.0	15.4	20.4	15.9	20.1	19.96
Protein (%)	17.63	15.92	15.13	16.23	12.43	15.468
Obesity (%)	16.2	14.9	23.5	35.6	68.5	31.74
Glucose	91	99	85	107	92	94.8
LDL	160	167	129	221	129	161.2
AAST	17	14	13	15	18	15.4

**Table 10:** False positive predictions (In. = Index).

Feature	In. 176	In. 211	In. 177	In. 299	In. 194	In. 316	In. 195	In. 203	In. 250	Mean
ALP	127	75	73	92	70	94	56	87	63	81.89
Creatinine	1.00	1.09	0.87	1.24	1.34	1.04	0.64	0.75	0.70	0.963
CRP	0.60	0.50	0.55	0.20	0.20	0.00	0.00	0.26	0.20	0.279
Vitamin D	12.7	26.0	23.3	36.9	26.7	15.7	30.5	25.8	12.2	23.31
DM	0	0	0	0	1	0	0	0	1	0.222
Height	177	179	165	172	176	172	163	153	169	169.56
Weight	78.6	95.8	64.0	78.8	116.1	96.6	68.5	74.7	71.3	82.71
ICW	25.4	29.0	22.4	23.9	32.8	28.2	19.6	17.2	23.0	24.17
Protein (%)	19.20	15.85	15.35	18.47	14.13	15.87	14.99	13.77	14.98	15.96
Obesity (%)	14.1	21.95	6.8	21.0	70.5	48.4	17.1	45.0	1.9	27.64
Glucose	110	92	88	101	118	122	98	108	284	115.67
LDL	149	127	147	125	88	153	140	101	79	123.22
AAST	26	16	21	17	17	21	24	29	17	21.00

**Table 11:** Ten generated counterfactuals showing changes in CRP, Vitamin D, AAST, and Gallstone Status for index 176.

Serial No	CRP	Vitamin D	AAST	Gallstone Status
1	0.6 → 12.8	12.7	26.0	0 → 1
2	0.6 → 14.7	12.7	26.0 → 144.9	0 → 1
3	0.6 → 6.5	12.7 → 20.4	26.0	0 → 1
4	0.6 → 8.2	12.7 → 25.0	26.0	0 → 1
5	0.6 → 15.2	12.7	26.0	0 → 1
6	0.6 → 3.0	12.7	26.0	0 → 1
7	0.6 → 6.6	12.7 → 20.8	26.0	0 → 1
8	0.6 → 12.7	12.7	26.0 → 126.0	0 → 1
9	0.6 → 10.8	12.7 → 31.8	26.0	0 → 1
10	0.6 → 13.8	12.7	26.0 → 136.0	0 → 1

**Table 12:** Ten generated counterfactuals showing changes in CRP, Vitamin D, AAST, and Gallstone Status for index 73.

Serial No	CRP	Vitamin D	AAST	Gallstone Status
1	0.63	10.9 → 18.1	17.0 → 60.4	1 → 0
2	0.63	10.9 → 36.2	17.0 → 124.0	1 → 0
3	0.63	10.9 → 37.9	17.0 → 129.9	1 → 0
4	0.63	10.9	17.0 → 51.6	1 → 0
5	0.63	10.9 → 39.7	17.0	1 → 0
6	0.63	10.9	17.0 → 47.8	1 → 0
7	0.63	10.9 → 28.8	17.0 → 98.0	1 → 0
8	0.63	10.9 → 41.6	17.0 → 143.0	1 → 0
9	0.63	10.9	17.0 → 112.3	1 → 0
10	0.63	10.9 → 37.0	17.0 → 126.9	1 → 0

**Table 13:** Five generated counterfactuals showing changes in CRP, Vitamin D, AAST, and Gallstone Status for index 208.

Serial No	CRP	Vitamin D	AAST	Gallstone Status
1	13.9 → 0.1	13.6	36.0	1 → 0
2	13.9 → 0.3	13.6	36.0	1 → 0
3	13.9 → 0.4	13.6	36.0	1 → 0
4	13.9 → 0.5	13.6	36.0	1 → 0
5	13.9 → 0.1	13.6 → 3.9	36.0	1 → 0

**Table 14:** Performance comparison (CV = Cross-validation).

SL	Author	Dataset Type	Performance
01	Bozdag et al. [5]	Image	Accuracy = 94.4 %
02	Wang et al [6]	Image	AUC = 0.995
03	Obaid et al. [7]	Image	Accuracy = 98%
04	Pang et al. [8]	Image	Accuracy = 86.5%
05	Esen et al. [10]	Tabular	Accuracy = 85.42%; Features No = 38
	**Our Proposed Frameworks**		
SL	**Frameworks**	**Dataset Type**	**Performance**
01	RF without CV	Tabular	Accuracy = 81.25%; F-score = 79.07% Precision = 85%; Recall = 73.91% Features No = 38
02	RF with CV	Tabular	Accuracy = 78.42%; F-score = 77.75% Precision = 80.01%; Recall = 75.75% Features No = 38
03	RF-SCSO without CV	Tabular	Accuracy = 79.17% F-score = 77.78% Precision = 79.55%; Recall = 76.09% Features No = 13
04	RF-SCSO with CV	Tabular	Accuracy = 78.32% F-score = 77.44% Precision = 80.36%; Recall = 75.01% Features No = 13

**Table 15:** Selected features Gallstone Positive vs. Gallstone Negative.

	Gallstone Positive (Class 0)				Gallstone Negative (Class 1)		
Type	CRP	Vitamin D	AAST		CRP	Vitamin D	AAST
Original Dataset	0.46	24.90	23.91		3.27	17.83	19.41
Wrong Prediction	0.279	23.31	21.00		0.718	16.626	15.4

## Data Availability

Dataset is available on UC Irvine Machine Learning Repository. Dataset Name: Gallstone. https://doi.org/10.1097/md.0000000000037258.
